# Pituitary Apoplexy: A Case Series

**DOI:** 10.7759/cureus.97380

**Published:** 2025-11-20

**Authors:** Dayanidhi Meher, Vishal Agarwal, Sambit Das, Arun Choudhury, Sandeep K Sahu, Devadarshini Sahoo, Binod Prusty, Bijay Das, Amogh S Chappalagavi, Sheenam Gupta

**Affiliations:** 1 Endocrinology, Diabetes and Metabolism, Kalinga Institute of Medical Sciences, Bhubaneswar, IND

**Keywords:** cabergoline, pituitary apoplexy, pituitary hemorrhage, pituitary macroadenoma, pituitary microadenoma, prolactinoma

## Abstract

Pituitary apoplexy (PA) is a rare but potentially life-threatening endocrine emergency, typically resulting from sudden-onset haemorrhage within the pituitary gland. If not promptly diagnosed and managed, it can lead to severe hormonal disturbances and neuro-ophthalmic complications with fatal consequences. Early recognition is crucial for improving patient outcomes. Certain conditions increase susceptibility to PA, including pre-existing pituitary adenomas (most commonly non-functioning), the postpartum period, diabetes mellitus, hypertension, sickle cell anaemia, and acute shock. Here, we describe five cases of PA, each with varied signs and symptoms, which underscores the clinical heterogeneity with which pituitary apoplexies can present. The first and second patients exhibited significant pituitary dysfunction and hyponatremia, necessitating steroid replacement and supportive care, leading to gradual recovery. The third patient presented with an incidental MRI finding of pituitary haemorrhage and remained hemodynamically stable without any evidence of hypopituitarism. The fourth case presented with cranial nerve palsies, while the fifth case, which presented as a prolactinoma, had apoplexy after starting cabergoline therapy. A high index of suspicion is essential for timely diagnosis. Emergency magnetic resonance imaging (MRI) of the sellar region is the gold standard for confirming the diagnosis. Rapid intervention, including appropriate hormonal replacement and neurosurgical evaluation, can be life-saving. This case series highlights the diverse clinical presentations of PA and outlines a structured approach to its management.

## Introduction

Pituitary apoplexy (PA) is a rare but potentially life-threatening clinical syndrome resulting from sudden haemorrhage or infarction within the pituitary gland, most commonly in the context of a pre-existing pituitary adenoma. The epidemiology of PA indicates a prevalence of approximately 6.2 cases per 100,000 population and an incidence of 0.17 episodes per 100,000 population annually [1,2]. Among patients with known pituitary adenomas, apoplexy occurs in up to 2-10% of cases, often as the initial manifestation of the tumor. While both genders can be affected, middle-aged men are slightly more predisposed, and the majority of cases present in the fifth or sixth decade of life [3].

Several mechanisms have been proposed to explain the pathogenesis of PA. Unlike the normal pituitary, which relies mainly on the portal system, adenomas often derive blood supply from the inferior hypophyseal artery, making it more vulnerable to systemic blood-pressure fluctuations and thereby predisposing to ischemia or bleeding [4,5]. Intrasellar pressure is also higher in adenomas, particularly in those presenting with PA, further compromising blood flow [6]. Tumor vasculature tends to be fragile and poorly organized, with reduced microvascular density, disrupted basal membranes, and lower VEGF expression, all of which may contribute to vascular instability [7]. Recognized precipitants include major surgery, anticoagulation, dynamic hormonal testing, dopamine or GnRH agonists, and head trauma, but up to 40% of cases occur without identifiable triggers [3].

Clinically, patients typically present with a constellation of acute symptoms: sudden onset of severe headache, visual disturbances (including visual field defects and ophthalmoplegia), altered mental status, and signs of hypopituitarism, most notably adrenal insufficiency. Nausea, vomiting, photophobia, and neck stiffness may mimic subarachnoid haemorrhage or meningitis [8]. The differential diagnosis includes subarachnoid haemorrhage, meningitis, cavernous sinus thrombosis, intracerebral haemorrhage, and other sellar or parasellar masses such as meningioma or craniopharyngioma. Hence, neuroimaging is essential to establish the diagnosis.

Diagnosis relies on magnetic resonance imaging (MRI), which is the modality of choice due to its high sensitivity in detecting haemorrhagic or ischemic changes within the pituitary. Computed tomography (CT) may identify acute haemorrhage but often misses subtle findings. Hormonal assays should evaluate anterior pituitary function, especially for cortisol, ACTH (adrenocorticotropic hormone), TSH (thyroid stimulating hormone), free T4, LH (luteinizing hormone), FSH (follicle-stimulating hormone), prolactin, and GH/IGF-1 (growth hormone/ insulin-like growth factor 1) axes [9].

The management of PA is multidisciplinary. Initial treatment focuses on hemodynamic stabilization and high-dose intravenous corticosteroids to prevent adrenal crisis. Surgical decompression via a transsphenoidal approach is indicated in patients with progressive visual deficits or deteriorating consciousness, whereas stable patients may be managed conservatively. Long-term complications include persistent hypopituitarism, visual impairment, and recurrence of apoplexy or tumor regrowth. Lifelong endocrinological follow-up is often necessary. Early diagnosis and timely management are crucial, as delayed treatment can lead to permanent visual loss or lifelong hormone deficiencies [10].

A better understanding of its varied clinical and biochemical presentations can help clinicians identify this condition earlier and improve outcomes. Most published literature originates from Western populations, while data from South Asian countries remain sparse. Few Indian case series have compared the biochemical, radiological, and clinical diversity of PA, or discussed outcomes across different management strategies [11]. Our series aims to address this gap by presenting five diverse cases of PA, spontaneous and drug-induced, managed in a tertiary endocrine unit. Through systematic description and comparative discussion, we aim to highlight diagnostic variability and management challenges in this uncommon but clinically significant condition.

## Case presentation

Case 1: pituitary apoplexy in a 67-year-old male

A 67-year-old male presented to the emergency department with a depressed sensorium following a two-day history of intense headache. He had been healthy prior to this presentation and had no history of similar episodes in the past. There was no associated fever or visual disturbance. He had no history of diabetes or hypertension but reported occasional headaches over the past two years, for which he took analgesics as needed.

On examination, his temperature was 37.1°C (98.8°F). He had a depressed sensorium, with a pulse rate of 104/min and blood pressure of 100/66 mmHg. Cardiovascular, respiratory, and abdominal examinations were unremarkable. Neurological assessment revealed a depressed level of consciousness with Glasgow coma scale score (GCS score) of 12/15 (E3V4M5). All cranial nerves were intact, and there were no focal neurological deficits. The plantar response was flexor bilaterally, and there were no signs of meningeal irritation. No visual field defects were noted (Table 1).

**Table 1:** Sequential clinical milestones (symptom onset to ED/OPD arrival, ED/OPD-to-imaging time), visual acuity and field defects, cranial nerve involvement, GCS, and Pituitary Apoplexy Scores for the five documented cases of pituitary apoplexy. Abbreviations: ED, emergency department; OPD, outpatient department; MRI, magnetic resonance imaging; OD, right eye; OS, left eye; ICU, intensive care unit; GCS, Glasgow Coma Scale

Parameter	Case 1	Case 2	Case 3	Case 4	Case 5
Symptom onset to ED/OPD arrival	2 days	6 days	5 days	3 days	3 days after cabergoline initiation
ED/OPD to imaging (MRI) (approximate time)	4 hours	3 hours	6 hours	3 hours	1 hour
Visual acuity	OD: 6/9, OS: 6/9	OD: 6/9, OS: 6/9	OD: 6/12, OS: 6/12	OD: 6/6, OS: 6/24	Could not be assessed due to low GCS
Visual fields	Confrontation: Normal	Confrontation: Normal	Confrontation: Bitemporal hemianopia	Confrontation: Bitemporal hemianopia	Could not be assessed due to low GCS
Cranial nerve involvement	None	None	None	Left 3rd nerve palsy	Could not be assessed due to low GCS
GCS at presentation	12/15	14/15	15/15	15/15	7/15
PAS (Pituitary Apoplexy Score)	2	2	4	4	Could not be assessed due to low GCS

Laboratory investigations showed a total leukocyte count of 8 × 10⁹ cells/L (normal range: 4.3-10.8 × 10⁹ cells/L) with neutrophilia. Haemoglobin was 110 g/L (normal range: 100-155 g/L), and the platelet count was 187 × 10⁹/L (normal range: 150-400 × 10⁹/L). Fasting plasma glucose was 4.3 mmol/L (normal range: <5.6 mmol/L). Liver function tests and lipid profile were within normal limits. Electrolyte and hormonal analysis (Table 2) revealed profound hyponatremia with a serum sodium of 103 mmol/L (normal range: 135-145 mmol/L) and a markedly low morning (8 AM) serum cortisol of 53 nmol/L (normal 8 AM cortisol: >275 nmol/L) along with hypogonadotropic hypogonadism.

**Table 2:** Clinical, biochemical, hormonal and radiological profile of the cases.

Cases	Case 1	Case 2	Case 3	Case 4	Case 5
Clinical profile					
Age	67 years	56 years	52 years	51 years	49 years
Sex	Male	Female	Female	Male	Male
Background medical history	None	None	None	None	Known case of prolactinoma.
Drug history	None	None	None	None	Cabergoline 0.5 mg/ week
Presentation	Sudden-onset headache, altered sensorium	Sudden-onset headache, altered sensorium	Insidious, fever, myalgia, occasional headache	Sudden onset headache, vomiting, 3^rd^ cranial nerve palsy	Sudden-onset headache, vomiting, altered sensorium
Radiological investigation					
Non-contrast CT head	-	-	-	-	Pituitary hemorrhage (Figure 5A)
MRI Brain	Pituitary microadenoma with haemorrhage (Figure 1)	Pituitary macroadenoma with haemorrhage (Figure 2)	Pituitary macroadenoma with haemorrhage (Figure 3)	Pituitary macroadenoma with haemorrhage (Figure 4)	Giant prolactinoma (macroadenoma) (Figure 5B)
Biochemical and hormonal profile					
Serum sodium (range: 135-145 mmol/L)	103 mmol/L	111 mmol/L	138 mmol/L	135 mmol/L	121 mmol/L
Serum potassium (range: 3.5-5.5 mmol/L)	4.0 mmol/L	3.7 mmol/L	4.2 mmol/L	4.1 mmol/L	3.8 mmol/L
Serum creatinine ( range: 44-97 μmol/L)	52 μmol/L	61.9 μmol/L	58 μmol/L	60 μmol/L	46 μmol/L
Serum prolactin (Male range: 3-20 ng/mL) (Female range: 3-30 ng/mL)	0.8 ng/mL	36.1 ng/mL	23.4 ng/mL	2.82 ng/mL	1386 ng/mL
Serum cortisol (range: >275 nmol/L)	53 nmol/L	26.8 nmol/L	515 nmol/L	14.1 nmol/L	100 nmol/L
Free T4 (range: 9-25 pmol/L)	6.57 pmol/L	5.24 pmol/L	15.5 pmol/L	11.6 pmol/L	5.14 pmol/L
TSH (range: 0.5-4.5 mIU/L)	0.94 mIU/L	3.82 mIU/L	3.4 mIU/L	0.034 mIU/L	0.729 mIU/L
Serum luteinizing hormone (LH) (Male range: 1.2-7.8 mIU/mL) (Post-menopausal range: 11.3 to 39.8 mIU/mL)	0.99 mIU/mL	1.68 mIU/mL	19.2 mIU/mL	0.63 mIU/mL	0.37 mIU/mL
Serum follicle-stimulating hormone (FSH) (Male range: 1.4-15.4 mIU/mL) (Post-menopausal range: 25.8 to 134.8 mIU/mL)	1.56 mIU/mL	5.11 mIU/mL	20.3 mIU/mL	2.02 mIU/mL	0.61 mIU/mL
Serum testosterone (Male range: 2.64-9.60 ng/mL)	0.02 ng/mL	-	-	0.03 ng/mL	2.03 ng/mL
Serum estradiol (Post-menopausal range: <30 pg/mL)	-	18.32 pg/mL	25 pg/mL	-	-
Serum insulin-like growth factor-1	70 ng/mL (normal range: 75-208 ng/mL)	82 ng/mL (normal range: 80-209 ng/mL)	128 ng/mL (normal range: 80-209 ng/mL)	68 ng/mL (normal range: 81-214 ng/mL)	100 ng/mL (normal range: 91-233 ng/mL)

**Figure 1:**
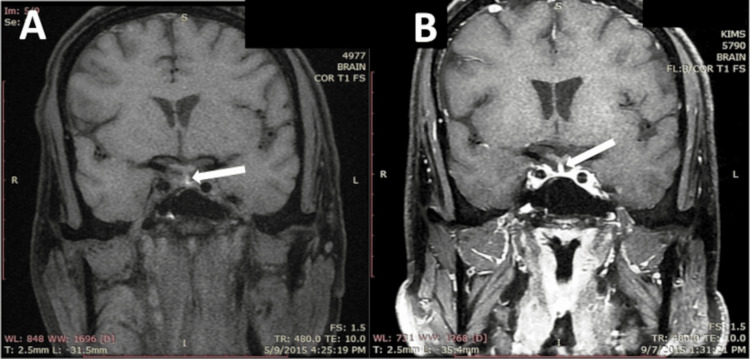
Case 1: (A) Coronal T1-weighted MRI (fat saturated sequence) shows a midline hyperintensity (arrow) that is seen at the junction of infundibular stalk with pituitary. This hyperintensity appears to be a hemorrhagic focus possibly occurring along the medial aspect of a hypointense pituiary mass (microadenoma) of size 0.6 cm x 0.7 cm x 0.8 cm (anteroposterior x transverse x craniocaudal) that is located at its right half (arrow). (B) Coronal T1-weighted MRI (fat saturated sequence) contrast image showing empty sella (arrow) after pituitary apoplexy during follow-up.

A non-contrast CT (NCCT) scan of the brain did not show any parenchymal abnormalities, as the pituitary was not the primary area of focus. However, due to the clinical suspicion of pituitary pathology, an MRI of the pituitary and hypothalamus was performed, which revealed a bulky right half of the pituitary with a differentially enhancing lesion containing a small hemorrhagic focus along its medial aspect, involving the stalk (Figure 1A). The patient was diagnosed with nonfunctioning pituitary adenoma (NFPA) with PA.

The patient was treated with intravenous fluids, hypertonic saline (3% NaCl), hydrocortisone 100 mg every eight hours, and levothyroxine 100 µg/day. His sensorium improved over the next two days, and serum sodium normalized by the third day. Hydrocortisone was tapered to a replacement dose on the fifth day and was started on testosterone replacement therapy. A follow-up MRI of the brain at four months showed an empty sella (Figure 1B). The patient was continued on hormone replacement therapies in view of persistent multiple pituitary hormone deficiencies during follow-up at three months.

Case 2: pituitary apoplexy in a 56-year-old female

A 56-year-old female presented to the emergency department with a five-day history of headache, followed by rapidly worsening altered sensorium over one day. There was no history of fever, trauma, or visual disturbances. She had no known history of diabetes or hypertension.

On examination, the patient was confused and slightly agitated (GCS 14/15). She was afebrile, with a temperature of 36.7°C (98.1°F). Her pulse rate was 102/min, and blood pressure was 102/60 mmHg. Cardiovascular, respiratory, and abdominal examinations were unremarkable. Neurological examination revealed no cranial nerve deficits or other focal neurological abnormalities, and the plantar response was equivocal. There were no signs of meningeal irritation. Visual fields were intact (Table 1).

**Figure 2:**
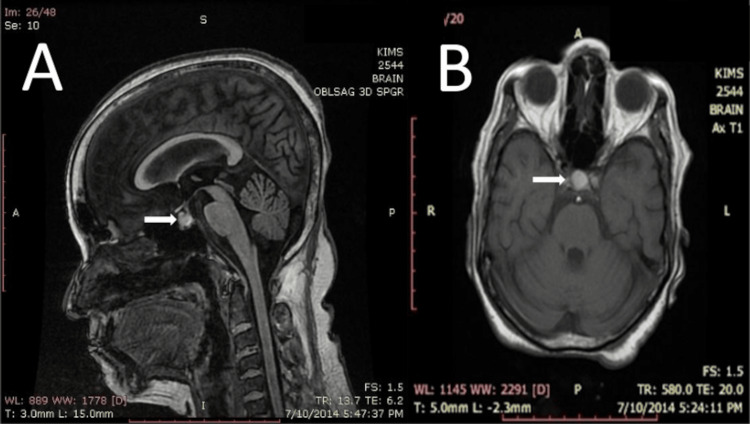
Case 2: (A) Sagittal T1-weighted (SPGR sequence) MRI shows a heterogeneously hyperintense anterior pituitary mass (arrow). (B) Corresponding axial T1-weighted MRI (SPGR sequence) image of the same patient shows a discrete rounded hyperintense mass (arrow) suggestive of haemorrhagic pituitary macroadenoma of size 0.7 cm x 0.9 cm x 1.1 cm (anteroposterior x transverse x craniocaudal). Abbreviations: SPGR, spoiled gradient recalled

Laboratory investigations revealed a hemoglobin level of 89 g/L (normal range: 120-155 g/L) and a total leukocyte count of 4.79 × 10⁹ cells/L (normal range: 4.3-10.8 × 10⁹ cells/L) with a normal differential count. Renal function was normal, with a serum urea level of 3.6 mmol/L (normal range: 2.5-7.1 mmol/L) and creatinine of 61.9 µmol/L (normal range: 45-90 µmol/L). Electrolyte analysis (Table 2) showed severe hyponatremia with a serum sodium of 111 mmol/L (normal range: 135-145 mmol/L) and a potassium level of 3.7 mmol/L (normal range: 3.5-5.5 mmol/L). Blood glucose levels were within normal limits, with a fasting plasma glucose of 4.2 mmol/L (normal range: <5.6 mmol/L) and a postprandial glucose of 7.5 mmol/L (normal range: <7.8 mmol/L). Hormonal assessment (Table 1) showed central hypothyroidism along with secondary adrenal insufficiency. MRI of the brain revealed a bulky pituitary gland with subacute hemorrhage, suggestive of PA, with a possible underlying Rathke’s cleft cyst (Figure 2).

The patient was treated with intravenous volume replacement, hypertonic saline (3% NaCl), intravenous hydrocortisone 100 mg every 8 hours, and levothyroxine 100 µg daily. She showed significant clinical improvement and regained full consciousness over three days.

Case 3: a mild presentation of pituitary hemorrhage in a 52-year-old woman

A 52-year-old woman was admitted to the medicine ward with a five-day history of fever, vomiting, and occasional headaches, along with generalized body aches. She had no history of diabetes or hypertension and had two children, with her last childbirth 21 years ago. Despite feeling unwell, she remained alert and oriented.

On examination, she was conscious and well-oriented, with a GCS score of 15. She was febrile, with a temperature of 39.3°C (102.7°F), a pulse rate of 114/min, and a blood pressure of 124/82 mmHg. Her cardiovascular, respiratory, and abdominal examinations were normal. Neurologically, all cranial nerves were intact, and she showed no signs of meningeal irritation. Her plantar response was flexor bilaterally. Visual field examination revealed bitemporal hemianopia (Table 1).

Routine blood tests, including a complete blood count, were normal. Tests for malaria and dengue were negative. Serum electrolytes (Table 2) were all within the normal range. Inflammatory markers, including ESR, were unremarkable, and her chest X-ray showed no abnormalities.

**Figure 3:**
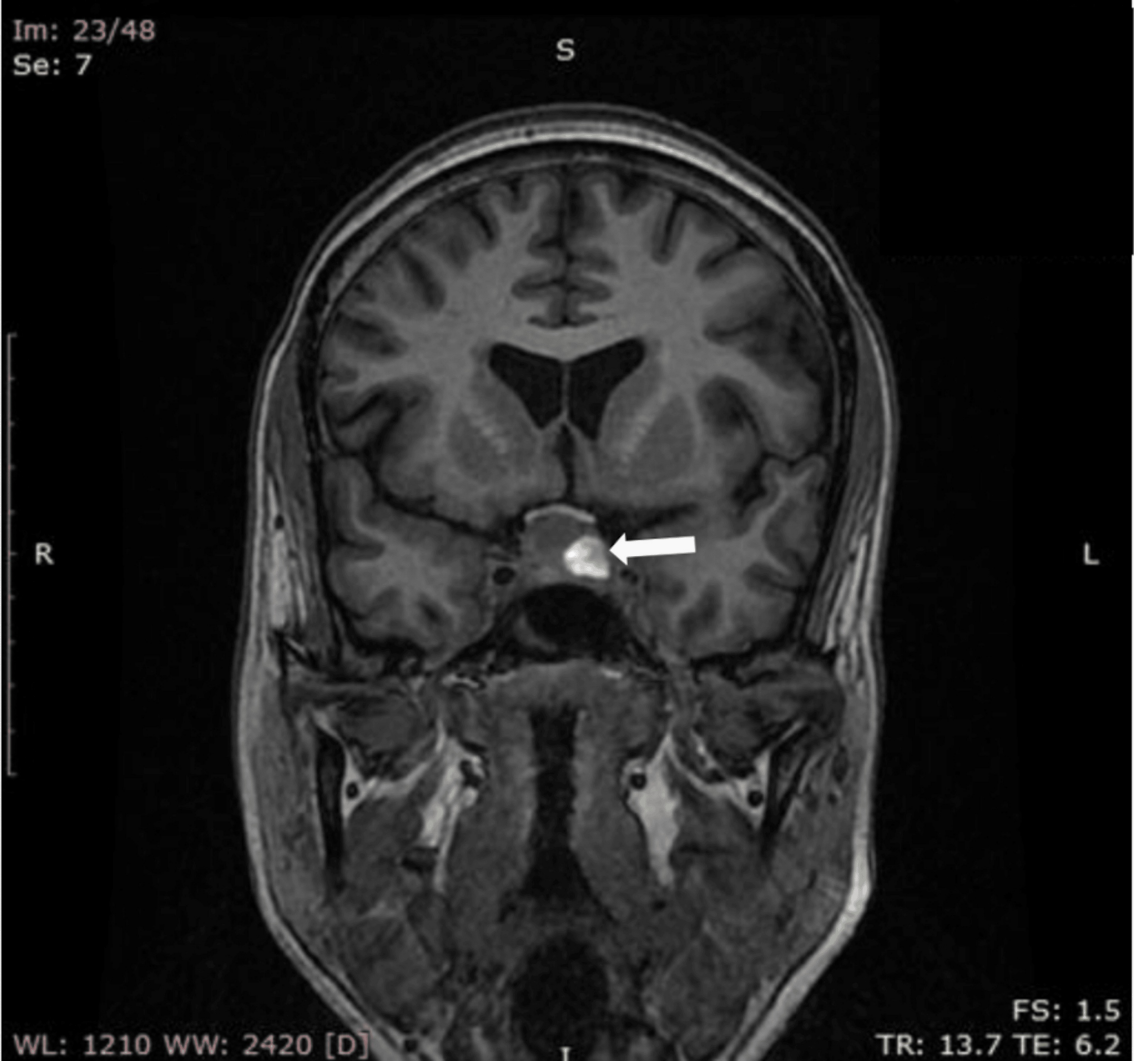
Case 3: Coronal T1-weighted MRI (SPGR sequence) shows a hypointense pituitary mass (macroadenoma) of size 1.1 cm x 1.5 cm x 1.6 cm (anteroposterior x transverse x craniocaudal) with a hyperintense focus (arrow) along the left lateral aspect that represents intratumoral haemorrhage. Optic chiasmal compression is also evident. SPGR: spoiled gradient recalled

Given her intermittent headaches, a MRI brain was performed, which unexpectedly revealed a pituitary haemorrhage within a pituitary macroadenoma, slightly abutting the optic chiasma (Figure 3). Further hormonal testing was conducted (Table 2), which showed a morning serum cortisol of 515 nmol/L (normal 8 AM cortisol: >275 nmol/L), a free T4 of 15.5 pmol/L (normal range: 9-25 pmol/L), and a TSH of 3.4 mIU/L (normal range: 0.5-4.5 mIU/L), suggesting normal adrenal and thyroid function. Gonadotropin levels were within the expected post menopausal range.

Since she remained stable and did not show any clinical signs of pituitary dysfunction, she was initially managed conservatively with supportive care and was later subjected to endoscopic transnasal trans-sphenoidal resection of pituitary tumor on day 4 of admission in view of visual field defects. After eight days in the hospital, she was discharged in good health, with reassurance and follow-up advice for monitoring her pituitary function.

Case 4: pituitary apoplexy in a 51-year-old male

A 51-year-old male presented with a three-day history of headache, vomiting, drooping of the left eyelid, and blurring of vision in the left eye. He had no history of diabetes, hypertension, or other comorbidities. On examination, he was alert and oriented with a GCS score of 15. His vital signs were stable. Neurological assessment revealed partial ptosis of the left eyelid with a dilated and sluggish left pupil. Visual acuity was 6/6 in the right eye and 6/24 in the left eye. The remaining cranial nerves were intact, and there were no focal neurological deficits (Table 1).

**Figure 4:**
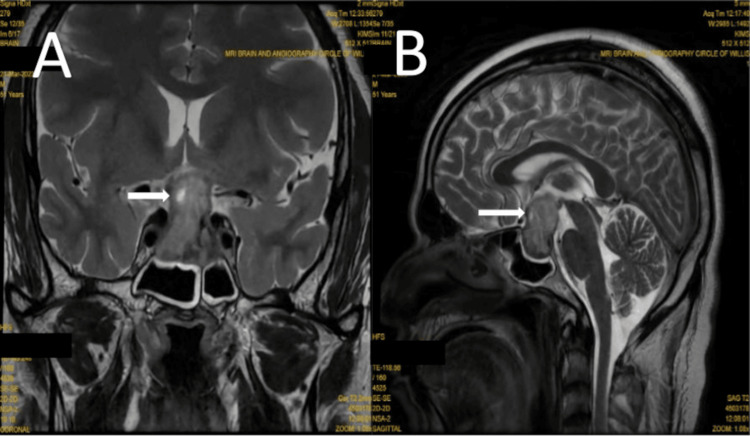
Case 4: T2-weighted MRI image showing pituitary apoplexy (arrow) in a macroadenoma of size 1.9 cm x 3.3 cm x 3.4 cm (anteroposterior x transverse x craniocaudal). (A) coronal section and (B) sagittal section.

An NCCT brain performed prior to admission showed a well-defined hyperdense lesion in the pituitary fossa with suprasellar extension, raising suspicion of a pituitary macroadenoma. Subsequent contrast-enhanced MRI (CEMRI) of the brain with angiography confirmed a pituitary macroadenoma measuring 1.9 cm × 3.3 cm × 3.4 cm (anteroposterior x transverse x craniocaudal) with internal hemorrhagic content, consistent with PA (Figure 4). Preoperative endocrine evaluation (Table 2) revealed central hypothyroidism, hypoadrenalism, and secondary hypogonadism due to pituitary insufficiency.

The patient underwent endoscopic transnasal trans-sphenoidal tumor decompression. Intraoperatively, a necrotic, firm, vascular tumor was visualized and successfully decompressed without cerebrospinal fluid (CSF) leakage. Postoperatively, the patient was extubated the following day and remained hemodynamically stable throughout his hospital stay. Postoperative (day 5) hormonal testing revealed persisting adrenal insufficiency, with an 8 AM cortisol level of 82 nmol/L (normal range: >275 nmol/L), necessitating continued steroid replacement therapy. By the time of discharge, the patient had shown significant improvement, with partial resolution of ptosis. He was hemodynamically stable and discharged on hydrocortisone, levothyroxine, and testosterone replacement therapy. This case highlights a classic presentation of PA with third nerve palsy, secondary hypoadrenalism, and hypogonadism, successfully managed with early surgical decompression and hormonal replacement therapy.

Case 5: pituitary apoplexy in a 49-year-old male with giant prolactinoma

A 49-year-old male, previously diagnosed with a prolactinoma, was started on cabergoline 0.25 mg twice weekly following markedly elevated serum prolactin levels (1386 ng/mL, normal range: 3.0-20 ng/mL). Three days after initiating treatment, he developed severe headache, visual impairment, vomiting, and altered sensorium, prompting emergency evaluation.

On admission, the patient was drowsy, with a GCS score of 7/15 (E2V2M3). His vital signs included blood pressure of 130/80 mmHg, pulse rate of 92/min, and random blood glucose of 8.6 mmol/L (normal range: <7.8 mmol/L). Neurological examination showed a right pupil of 3 mm reactive to light and a left pupil of 2 mm reactive to light, with severely depressed sensorium (Table 1).

**Figure 5:**
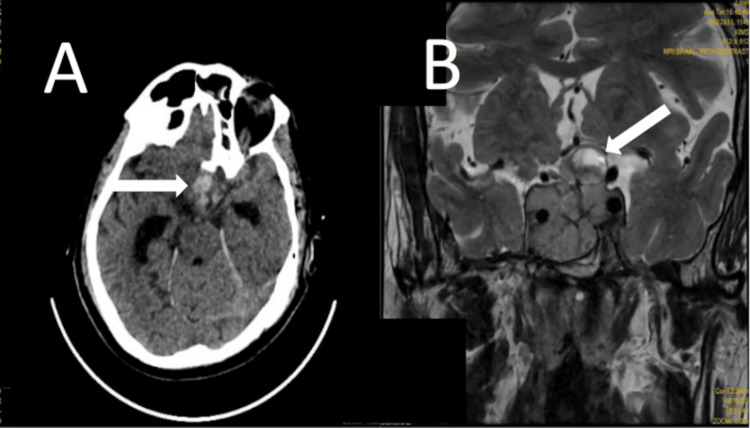
Case 5: (A) Computed tomography scan (axial section) done in the emergency department showing pituitary haemorrhage (arrow) after starting cabergoline therapy. (B) MRI T2-weighted image (coronal section) showing giant prolactinoma (arrow) with pituitary apoplexy.

An NCCT head was done at emergency department, confirmed PA with lobulated heterodense hemorrhagic changes (arrow) (Figure 5A). MRI brain revealed a pituitary macroadenoma with PA (arrow) (Figure 5B). 

The patient was initially managed in the neurosurgical intensive care unit (ICU) due to low GCS and required intubation due to respiratory distress. After stabilization, the patient underwent transnasal transsphenoidal (TNTS) tumor excision. Intraoperative findings included a grayish-white, soft-to-firm tumor without CSF leakage, and the sellar floor was reconstructed using a tensor fascia lata graft.

Postoperatively, the patient remained on prolonged ventilatory support, necessitating a tracheostomy. The postoperative course was complicated by episodes of bradycardia, tachycardia, severe hypertension, and fluctuating sodium and potassium levels, requiring multidisciplinary management. Due to progressive hydrocephalus, a ventriculoperitoneal (VP) shunt was placed at Frazier’s point on post-operative day 20. The patient remained in the ICU for an extended period due to persistent metabolic and hemodynamic instability. He was successfully decannulated on day 30 postoperative day and later transferred to the neurosurgical ward for further rehabilitation.

By the time of discharge, the patient was hemodynamically stable but remained bedridden, requiring nasogastric tube feeding and urinary catheterization. He was discharged on levetiracetam, thyroxine, antihypertensives, and supportive therapy, with physiotherapy recommendations. Regular follow-ups were scheduled in the neurosurgery and endocrinology outpatient departments.

This case highlights a challenging presentation of recurrent pituitary macroadenoma with apoplexy, triggered by dopamine agonist therapy (cabergoline), leading to hydrocephalus and requiring TNTS surgery, VP shunting, and prolonged intensive care support.

## Discussion

PA is a rare but potentially life-threatening endocrine emergency caused by hemorrhage or infarction of the pituitary gland. PA most often arises in the context of a pituitary adenoma, with nearly 80% of cases occurring in previously unrecognized tumors. It is more frequently observed in macroadenomas, particularly NFPAs, which represent the most common subtype [3]. PA has also been described in giant adenomas, and in these, approximately 60% were already diagnosed prior to the apoplectic event [12]. Among hormone-secreting tumors, prolactinomas are most frequently implicated [13]. Several risk factors have been associated with PA, including surgical interventions, use of anticoagulation, medical therapy for pituitary tumors, dynamic pituitary testing, hormonal treatment, and head trauma. Nonetheless, in 10-40% of cases, no identifiable predisposing factor is found [3,9]. Dopamine agonist therapy, such as bromocriptine, has also been linked to PA in some cases [14].

Multiple mechanisms have been proposed to explain the pathogenesis of PA. Pituitary adenomas exhibit high metabolic activity, demonstrated by increased uptake of [18F]-fluorodeoxyglucose and [11C]-L-methionine on PET imaging, making them particularly vulnerable to any reduction in blood or nutrient supply [15]. Unlike the normal pituitary, which is mainly nourished by the hypophyseal portal system, adenomas often derive their blood supply from the inferior hypophyseal artery [16]. This arterial dependence renders them more susceptible to fluctuations in systemic blood pressure and flow, potentially triggering ischemia or hemorrhage. In addition, intrasellar pressure is typically elevated in patients with pituitary tumors and is further increased in those presenting with apoplexy [17,18].

The hallmark presenting feature of PA is a sudden, severe headache, usually frontal or retro-orbital, reported in 80-90% of cases [19]. Cranial nerve palsies involving the oculomotor, trochlear, or abducens nerves occur in 39-52% of cases, with third nerve involvement being most common. Altered consciousness is observed in up to 42% of patients [20]. Associated symptoms such as nausea, vomiting, and photophobia may mimic meningitis [21,22].

Because the clinical presentation can overlap with other acute central nervous system disorders, differential diagnoses include meningitis, hypophysitis, subarachnoid hemorrhage, cerebral infarction, cavernous sinus thrombosis, and carotid artery dissection. While most patients present within 24-72 hours of the event, some come to medical attention later, which has implications for interpreting imaging findings.

Endocrine dysfunction is common, reflecting the presence of an underlying adenoma. Hypopituitarism is frequently identified at presentation, with secondary adrenal insufficiency reported in 45-70% of cases, often associated with hyponatremia, hypotension, or hypoglycemia [3,22,23]. Central hypothyroidism occurs in 35-70%, while hypogonadism is diagnosed in about 60% of men and 50-75% of women. Growth hormone deficiency is rarely evaluated acutely, so its prevalence is uncertain. Prolactin abnormalities may be seen, either hyperprolactinemia due to stalk effect or hypoprolactinemia from pituitary damage. Diabetes insipidus is uncommon but may occur in a small subset [22]. In addition, if the underlying tumor is hormonally active, symptoms of hormonal excess may precede the apoplectic event.

The timing of imaging in suspected PA is critical, as the radiological appearance evolves with the biochemical transformation of hemoglobin and subsequent blood degradation. CT is often the preferred first-line modality in the hyperacute phase (<6 hours from symptom onset), as fresh blood usually appears hyperdense. However, in some hyperacute cases blood may be isodense, and ischemic changes may be difficult to appreciate. With time, blood density gradually decreases until it approximates that of water.MRI provides greater sensitivity and anatomical detail but interpretation depends on the stage of hemoglobin breakdown. In the hyperacute phase, blood with deoxyhemoglobin is typically isointense on T1 and hyperintense on T2. Within 24-48 hours, intracellular methemoglobin accumulates, leading to T1 hyperintensity, which may persist for one to four weeks. As hemosiderin deposition occurs beyond two to four weeks, the T1 signal diminishes. On T2-weighted sequences, signal intensity is initially bright (hyperacute), becomes dark during the acute and early subacute phases (one to seven days), then brightens again with extracellular methemoglobin formation (seven to 28 days). In the chronic phase, hypointensity reappears due to hemosiderin deposition. Mixed signal intensities may produce fluid-fluid levels, reflecting coexisting stages of blood degradation [24].

PA often results in acute and profound anterior pituitary hormone deficiencies, which can cause significant electrolyte disturbances and hemodynamic compromise. Initial management therefore focuses on resuscitation, correction of electrolyte imbalances, and stabilization of the patient. The most urgent concern is adrenal insufficiency, followed by thyroid hormone deficiency. Empirical glucocorticoid therapy should be initiated without delay, typically intravenous hydrocortisone 200 mg per 24 hours, either as a continuous infusion or in divided doses of 50 mg every six hours [25]. If thyroid hormone replacement is required, it should only be introduced after glucocorticoid therapy has been established to avoid precipitating adrenal crisis.

Beyond acute stabilization, the optimal long-term management strategy is less clearly defined. Evidence has compared surgical approaches (microscopic vs. endoscopic transsphenoidal resection), timing of surgery (early vs. delayed), and outcomes of surgical versus conservative management, but results remain heterogeneous [26,27].

In our cases (Table 2), the first and second patients exhibited significant pituitary dysfunction and hyponatremia, necessitating steroid replacement and supportive care, leading to gradual recovery. The third patient, with an incidental MRI finding of pituitary hemorrhage, remained hemodynamically stable without evidence of hypopituitarism and was managed by surgical intervention in view of visual field defects. The fourth case presented with cranial nerve palsies, while the fifth case presented with apoplexy after starting cabergoline therapy.

To place our findings in context, Table 3 compares the clinical and radiological profile of our patients with those reported in major series on PA [11,13,23]. 

**Table 3:** Comparison of our case series with previously published data on pituitary apoplexy.

Parameter	Present Series (n = 5)	Sibal et al., 2004 (n = 45)	Bujawansa et al., 2014 (n = 55)	Ragate et al., 2024 (n = 93)
Study design	Retrospective, tertiary endocrine unit (India)	Retrospective, tertiary neurosurgical unit (United Kingdom)	Single-center retrospective (United Kingdom)	Retrospective, tertiary endocrine-neurosurgery center (India)
Mean age (years)	55 (49–67)	49 (16-72)	52.4 (14-78)	40 (18.5-74)
Sex ratio (M:F)	1.5:1	1.6:1	1.7:1	1.5:1
Predisposing factors	Hypertension, Cabergoline therapy	Hypertension, major surgery, aspirin therapy	Hypertension, aspirin, anticoagulation, major surgery	Cabergoline use, COVID-19 infection requiring hospitalization, dengue shock syndrome
Common presenting symptoms	Headache (100%), altered sensorium (60%), visual field defect (2 cases)	Headache (96%), nausea and vomiting (78%), ophthalmoplegia (51%), visual field defect (48%)	Headache (87%), diplopia (47.2%), visual field defect (36%)	Headache (78.5%), visual field defect 30.1%), vomiting (47%), altered sensorium (17%)
Management	3 surgical (TSS), 2 conservative	60% surgical, 40% conservative	55% surgical, 45% conservative	53.8% surgical, 46.2% conservative
Unique features / Contribution	Cabergoline-induced apoplexy	First large UK series defining clinical spectrum	Emphasized surgical timing and outcomes	Largest Indian cohort; functioning and non-functioning adenomas analyzed together

The demographic characteristics and mean age at presentation in our series were comparable to those reported by Sibal et al., Bujawansa et al., and Ragate et al., with a slight male predominance. The frequency of precipitating factors such as pituitary adenoma or hypertension was also consistent with previous studies. However, the proportion of cases presenting with visual field defects were higher in our cohort. This case series adds to the limited data from Indian centers on PA, particularly highlighting the clinical spectrum and management outcomes across both functioning and non-functioning adenomas. By systematically comparing our findings with major published cohorts, it bridges the regional gap in understanding presentation patterns and treatment decisions in resource-limited settings.

## Conclusions

PA, although rare, remains a true endocrine and neurosurgical emergency that demands prompt recognition and timely intervention. Through the presentation of these five diverse cases, this series underscores the protean nature of the syndrome, which can range from mild headache to sudden visual loss and life-threatening adrenal insufficiency.The variability observed reinforces the importance of maintaining a high index of suspicion, particularly in individuals with known pituitary adenomas or established risk factors. While imaging, particularly MRI, is indispensable for diagnosis, clinical suspicion remains paramount, especially in patients with known pituitary adenomas or predisposing risk factors. Management must be individualized, balancing the need for emergent surgical decompression in those with deteriorating neurological function against conservative therapy in stable patients.

However, the findings in this case series are primarily descriptive, and the absence of long-term follow-up metrics limits the ability to draw firm conclusions regarding comparative outcomes of different management strategies. Future studies incorporating standardized outcome measures, structured follow-up, and detailed surgical documentation including intraoperative imaging are needed to strengthen evidence-based recommendations for the optimal care of patients with PA.
